# Gene Decay in *Shigella* as an Incipient Stage of Host-Adaptation

**DOI:** 10.1371/journal.pone.0027754

**Published:** 2011-11-16

**Authors:** Ye Feng, Zhe Chen, Shu-Lin Liu

**Affiliations:** 1 Genomics Research Center, Harbin Medical University, Harbin, China; 2 National Clinical Research Base,Zhejiang Hospital of Traditional Chinese Medicine, Hangzhou, China; 3 Department of Microbiology, Peking University Health Science Center, Beijing, China; 4 Department of Microbiology and Infectious Diseases, University of Calgary, Calgary, Canada; University of Wyoming, United States of America

## Abstract

**Background:**

Many facultative bacterial pathogens have undergone extensive gene decay processes, possibly due to lack of selection pressure during evolutionary conversion from free-living to intracellular lifestyle. *Shigella*, the causative agents of human shigellosis, have arisen from different *E. coli*-like ancestors independently by convergent paths. As these bacteria all have lost large numbers of genes by mutation or deletion, they can be used as ideal models for systematically studying the process of gene function loss in different bacteria living under similar selection pressures.

**Methodologies/Principal Findings:**

We compared the sequenced *Shigella* genomes and re-defined decayed genes (pseudogenes plus deleted genes) in these bacteria. Altogether, 85 genes are commonly decayed in the five analyzed *Shigella* strains and 1456 genes are decayed in at least one *Shigella* strain. Genes coding for carbon utilization, cell motility, transporter or membrane proteins are prone to be inactivated. Decayed genes tend to concentrate in certain operons rather than distribute averagely across the whole genome. Genes in the decayed operon accumulated more non-synonymous mutations than the rest genes and meanwhile have lower expression levels.

**Conclusions:**

Different *Shigella* lineages underwent convergent gene decay processes, and inactivation of one gene would lead to a lesser selection pressure for the other genes in the same operon. The pool of superfluous genes for *Shigella* may contain at least two thousand genes and the gene decay processes may still continue in *Shigella* until a minimum genome harboring only essential genes is reached.

## Introduction

Bacterial genomes are usually tightly packed with protein-coding genes and other functional elements. From the evolutionary point of view, a bacterial genome stays at a general balance in terms of genetic material gains and losses. On the one hand, it receives exogenous DNA in order to acquire extra fitness traits for better adapting to the external environment, and on the other hand the genome itself is undergoing a shrinking process, mainly in the forms of pseudogenization and deletion. Supporting evidence includes the finding that the majority of pseudogenes are newly-born genes and the derived young pseudogenes may be expelled out of genome soon [1], [2]. Direct deletion can also occur to throw out DNA fragments of various sizes [3].

When bacteria evolve from free-living to an intracellular lifestyle, a balance between DNA flowing in and out to maintain a certain genomic size may vanish. In this process, some bacterial pathogens may accumulate a large number of pseudogenes, such as *Mycobacterium leprae* [4], which has half of its genes inactivated, and some others may even get rid of all non-essential genes to keep a minimum genome, such as *Mycoplasma genitalium* [5]. Perhaps the most important cause leading to genome shrinking is natural selection: genes that no longer make significant contributions to bacterial fitness may subject to decay (including pseudogene formation and complete deletion). Genetic drift is another important factor [6], [7], [8]. Especially for some highly pathogenic bacteria, which typically require only small inocula to elicit infection in their host, the effective population size is dramatically reduced. Such extreme population bottleneck greatly diminishes the efficacy of selection, under which even beneficial genes are prone to be eliminated. In addition, loss of certain genes will sometimes contribute to fitness, so the bacteria would tend not to express these genes. Such “adaptive loss” has been reported in *Shigella*. For example, expression of *cadA* in *Shigella* would inhibit the enterotoxin activity, therefore reducing the virulence. So this gene is usually inactivated in *Shigella* [9].

*Shigella* are human-restricted pathogens that are highly contagious. According to the traditional taxonomy, the *Shigella* genus consists of four species: *S. dysenteriae*, *S. flexneri*, *S. boydii* and *S. sonnei* [10]. However, modern genotyping methods, especially comparative genomic studies, have revealed that the four *Shigella* species are phylogenetically interwoven with the *E. coli* lineages, suggesting that distinct ancestral *E. coli* lineages developed into similar pathogens through convergent evolutionary processes by acquisition of common pathogenic traits [11], [12], [13]. Extensive gene decay has been documented in representative strains of all *Shigella* species [14], [15], [16], [17] and this process occurs at a much faster speed in *Shigella* than in typical *E. coli* [18], possibly reflecting a key series of evolutionary events in the conversion of *Shigella* from commensals into intracellular pathogens.

In this study we used *Shigella* as the model to investigate key issues about bacterial gene decay, including how convergent gene decay occurs in the process when bacteria of different origins adapt to the same biological niche, and how many superfluous genes will eventually be decayed. Different from previous studies, this study is characteristic of two aspects. First, we defined convergent pseudogenization as different inactivation mutations affecting the same protein. As a result, the majority of pseudogenes shared by different *Shigella* species belong to the convergent instead of ancestral pseudogene category. Second, we classified genes into decayed genes, intact genes in the decayed operons and the rest genes. In addition to demonstrating the existence of genome shrinking process as well as the lesser selection pressure combined with low expression of decayed genes, this operon-based analysis also delimits the minimal number of potentially superfluous genes.

## Results and Discussion

### Identification of decayed genes and genomic reduction of *Shigella*

**Figure 1:**
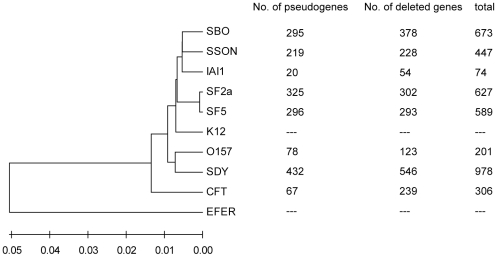
Phylogenetic tree of *E. coli*/*Shigella* strains and the number of decayed genes. The maximum likelihood tree was constructed by using the concatenated nucleotide sequences of 2500 orthologs conserved in all strains. K12 was used as the reference for identifying pseudogenes in the other strains; EFER was used as the outgroup for constructing the phylogenetic tree.

We used the genome of *Escherichia coli* K12 MG1655 (abbr. K12) as the reference sequence to identify gene loss in *Shigella*. The K12 genome has two special advantages for use as the reference: its vast sequence similarity with the *Shigella* strains and its largely experiment-based annotation [19]. Especially important is the fact that the gene boundaries of many CDSs of K12 have been verified by experiments, which would greatly facilitate pseudogene identification. The *Shigella* strains analyzed included *S. boydii* Sb227 (SBO), *S. dysenteriae* Sd197 (SDY), *S. flexneri* 2a 301 (SF2a), *S. flexneri* 5 8401 (SF5), and *S. sonnei* Ss046 (SSON). We also included *E. coli* O157:H7 EDL933 (O157), *E. coli* CFT073 (CFT073) and *E. coli* IAI1 (IAI1) in this study for comparison with *Shigella*, because the three strains as well as K12 belong to different *E. coli* phylogroups and therefore can represent a broad range of *E. coli* sublineages [20]. To reveal the genetic relationships among these strains, we built a phylogentic tree based on all conserved genes among them (Figure 1).

As expected, all *Shigella* strains had greatly more genes decayed (including both pseudogene formation and deletion) than *E. coli* (Figure 1; Table S1). Of particular interest, the numbers of decayed genes differ considerably among the *Shigella* species. For instance, SDY had nearly twice as many genes decayed as SSON, suggesting that SDY was the earliest *Shigella* lineage to become adapted to the human host. A total of 85 genes decayed in all five *Shigella* strains analyzed (Table S2), providing candidates for further scrutiny about possible positive selection of their absence. The previous reported gene *cadA* [9] was also in this list.

The Pearson correlation coefficient between the numbers of pseudogenes and deleted genes is close to 0.9, suggesting that pseudogenization and deletion occurred simultaneously. Some other findings also support this hypothesis. The two *S. flexneri* strains have diverged very recently. Despite the short divergence time, SF2a and SF5 each have 17 and 14 strain-specific deleted genes, respectively; meanwhile, they each have 14 and 10 strain-specific truncated pseudogenes (Table S1). Thus genomic reduction may not necessarily have started with pseudogenes formation first followed by deletion; direct deletion also seems to be a common event in the genomic evolution of bacteria.

### Convergent gene decay among *Shigella* species

Shared pseudogenes among the *Shigella* strains account for nearly one quarter of all pseudogenes in each strain (Table S1). We further classified the shared pseudogenes into three groups based on the inactivation causes. The first group is ancestral pseudogenes, which have the same inactivation mutations among the compared bacteria and therefore have probably been inherited from their last common ancestor. Only a minority of shared pseudogenes can be assigned to the ancestral situation in all comparisons. The only exception was with SF2a and SF5, which share most of their ancestral pseudogenes. They belong to the same *S. flexneri* species and therefore should have diverged not long ago.

The second group is convergent pseudogenes, which have inactivation mutations affecting different sites of the same gene among the bacteria and therefore have probably been formed after the divergence of the bacteria. Most of the shared pseudogenes belong to the convergence situation (Table S1), consistent with the hypothesis that *Shigella* lineages have developed from multiple origins (which is also indicated by the phylogenetic trees; see Figure 1). During their convergent paths to adapt to the same biological niche, a common set of genes would become superfluous, so the convergent pseudogenes should reflect the common superfluous functions.

The third group is recombinant pseudogenes, which have the same inactivation mutation among the compared strains but the mutation conflicts with the phylogenetic relationships. For example, SF2a and SBO shared the same pseudogene but the same gene remained intact in SF5 (a member of the same species as SF2a) and in other strains. Then the most plausible explanation is that such a pseudogene must have resulted from recombination that copied the mutation from SBO to SF2a. A total of 27 putative recombinant pseudogenes were identified, and 14 of them are found between *S. boydii* and *S. flexneri* (Table S3). The recombination rate in *Shigella* was in fact quite low: 0.013 for the five *Shigella* strains as compared to 0.056 for the four *E. coli* strains (see Text S1). In the real world, it should be quite unlikely that two different *Shigella* species might infect the same person at the same time. Consequently, recombination should not be considered as a major route of pseudogene formation as that between *Salmonella typhi* and *Salmonella paratyphi* A, two human-restricted *Salmonella* species [21].

Relatedness among SDY and O157 is quite intriguing. The two strains were clustered at the same branch of the phylogenetic tree as shown in Figure 1. It was once hypothesized that the ancestor of the two bacterial lineages had headed on the evolutionary paths toward becoming *Shigella*, but O157 lost the *Shigella* virulence plasmid and went back to a lower rate of gene loss [18]. In our analysis, the two strains share 30 pseudogenes, only one of which belongs to the ancestral category (Table S4), providing evidence against the above hypothesis. Here we prefer an alternative hypothesis: they were living in a common environment for a certain time period after their divergence, and the environment facilitated formation of the convergent pseudogenes.

### The pool of superfluous genes for *Shigella*

As many as 1456 genes have decayed through pseudogenization or deletion in one or more of the five *Shigella* strains analyzed (Table S2). The extensive gene decay, involving mostly the convergent evolutionary paths, suggests that these genes are superfluous (at least non-essential) functionally in the *Shigella* species. An interesting question is how large the pool of superfluous genes might be or whether there would be an end of the gene decaying process.

We categorized the 1456 genes by function using the COG and GenProtEC classification systems. Analyses by the two classification systems led to common conclusions: genes coding for carbon utilization, cell motility, transporter or membrane proteins are prone to be inactivated, whereas those responsible for maintenance of basic cellular metabolism and structure tend to resist inactivation (Chi square test, p value < 0.001; Table 1). This suggests that the reduction process will at least have a threshold, namely ending up with a minimum genome that contains only essential genes like *Mycoplasma genitalium* [5].

**Table 1:** Differential functional assignment between decayed genes and intact genes.

Database	Description	Type
COG	Carbohydrate transport and metabolism (G)	p
COG	Cell motility (N)	p
COG	Posttranslational modification, protein turnover, chaperones (O)	p
COG	Intracellular trafficking and secretion (U)	p
COG	Coenzyme transport and metabolism (H)	r
COG	Translation (J)	r
COG	Replication, recombination and repair (L)	r
GenProtEC	Carbon compound utilization (1.1)	p
GenProtEC	Electrochemical potential driven transporters (4.2)	p
GenProtEC	Motility (5.3)	p
GenProtEC	Membrane (6.1)	p
GenProtEC	Flagellum (6.4)	p
GenProtEC	Pilus (6.5)	p
GenProtEC	Outer membrane (7.4)	p
GenProtEC	Building block biosynthesis (1.5)	r
GenProtEC	DNA related (2.1)	r
GenProtEC	Protein related (2.3)	r
GenProtEC	Cell division (5.1)	r
GenProtEC	Peptidoglycan (6.2)	r
GenProtEC	Ribosome (6.6)	r
GenProtEC	Cytoplasm (7.1)	r

Note: Type ‘p’ indicates that genes of this functional category are prone to be inactivated; Type ‘r’ indicates that genes of this functional category are resistant to be inactivated.

**Figure 2:**
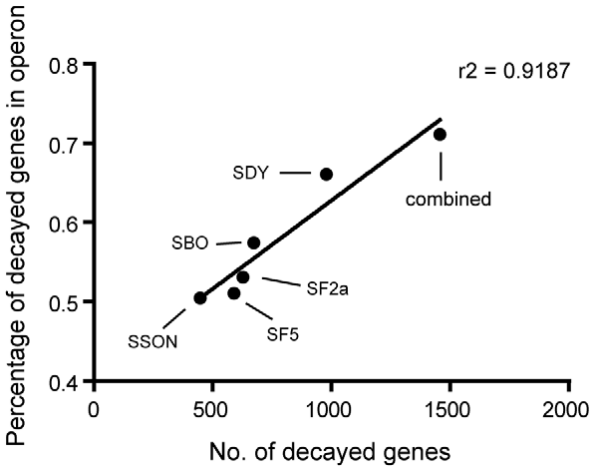
Correlation between number of decayed genes and percentage of decayed genes in operon. The dot “combined” represents all decayed genes in the five *Shigella* strains and the corresponding percentage of decayed genes in operon.

In bacteria, the basic functioning genetic unit is the operon, which contains a cluster of genes under the control of the same set of regulatory signals. Therefore, the inactivation of one gene may result in functional relaxation on the other genes within the same operon, starting the gene decay process. We searched for the operons in which the decayed genes are located with the premise that all genes in these “decayed operons” would be eventually eliminated. Meanwhile, we calculated the percentage of decayed genes over total genes within the operon for each strain. This index is highly correlated with the number of decayed genes (Figure 2). In other words, decayed genes are indeed more concentrated within certain operons rather than being scattered averagely in the whole genome. The 1456 decayed genes were located in 832 operons, which contain a total of 2048 genes (Table S2). We believe that this is the minimum size of the superfluous gene pool.

### Functional relaxation and low expression of pseudogenes

**Figure 3:**
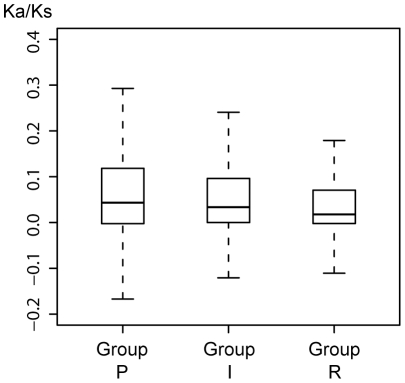
Comparison of Ka/Ks between different gene groups in SDY. The Ka/Ks was adjusted following **Methods**. Group P, Pseudogenes; Group I, the Intact genes in the decayed-operons; Group R, the Rest functional genes in the genome.

We hypothesized that pseudogenization would accelerate the accumulation of non-synonymous mutations in the pseudogenes as well as intact genes in the decayed operons (operons containing decayed genes). So these genes were expected to exhibit a higher Ka/Ks value in comparison with the rest genes. To look into this, we classified genes into three categories: Pseudogenes (Group P), the Intact genes in the pseudo-operons (Group I) and the Rest functional genes in the genome (Group R). We found that in SDY, the Ka/Ks values of group P and group I are significantly higher than group R, whereas between group P and group I no significant difference was detected (Kruskal-Wallis test with Dunn's post test, p<0.01; Figure 3). This result demonstrated the accuracy of operon-based classification and, in the meantime, showed the synchronous evolving speed for the genes in the same operon: the whole operon would decay immediately once one of its genes becomes inactivated, followed by relaxed selection pressure on the rest genes in the same operon. In other *Shigella* strains, no significant difference was found among the three groups. Given that SDY had undergone the most extensive gene decay and therefore might be the earliest *Shigella* lineage to become adapted to the human host, we presume that the other *Shigella* species have not had enough time yet for accumulating non-synonymous mutations like SDY.

**Figure 4:**
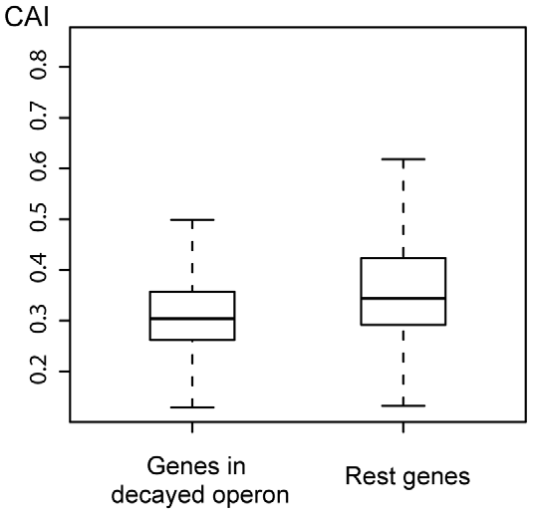
Comparison of CAI between genes in decayed operon and the rest genes in the genome.

Will pseudogenized genes be expressed and, if yes, how much? The relationship between pseudogenization and expression levels may involve two questions. The first is whether genes of low expression are more prone to become pseudogenized, and the second is whether genes would decrease the expression levels after pseudogenization. For the first question, we compared the Codon Adaptation Index (CAI) between the genes in decayed operon and the rest genes, since this index has long been used to predict expression levels [22], [23]. The result showed that the former did have lower CAI values than the latter (Unpaired t test, p value < 0.001; Figure 4). It is likely that genes of low expression are only occasionally expressed when the bacteria encounter certain environmental stimuli. Once living in a relatively stable environment, loss of these genes would not cause obvious adverse effects and therefore gene decay may ensue.

For the second question, we resorted to microarray-based transcription data. A total of eight microarray samples that measured the expression profile of *Shigella* were deposited in NCBI GEO database. The original purpose of these microarray experiments covers comparison between conditions under different temperatures, or comparison between in-vivo and in-vitro conditions, or comparison between before and after curation of virulence plasmid, etc. Nearly all these samples exhibited lower expression of genes in decayed operons than the rest genes (Kruskal-Wallis test with Dunn's post test, p value < 0.01). This phenomenon can be explained as the following: the bacteria try to avoid extra investment of resource and energy on the decayed genes. Additionally, malfunctioning proteins encoded by pseudogenes may be detrimental to the bacteria by interacting with other pathways, e.g., by losing specificity or competing for substrates [1]. However, the pseudogenes and the intact genes within the decayed operons have similar expression, demonstrating the read-through transcription of both intact genes and pseudogenes together within the same operon.

## Materials and Methods

### Genomic sequences

The genomic sequences analyzed in this study were taken from NCBI Genbank database, including *Shigella sonnei* Ss046 (CP000038), *S. boydii* Sb227 (CP000036), *S. dysenteriae* Sd197 (CP000034), *S. flexneri* 2a 301 (AE005674), *S. flexneri* 5 8401 (CP000266), *Escherichia coli* K12 MG1655 (U00096), *E. coli* O157:H7 EDL933 (AE005174), *E. coli* CFT073 (AE014075), *E. coli* IAI1 (CU928160) and *E. fergusonii* ATCC35469 (CU928158).

### Construction of orthologs and Identification of decayed genes

The coding sequences (CDSs) from *E. coli* K12 MG1655 were mapped to query genomes for ortholog identification by using NCBI Basic Local Alignment Search Tool (BLAST), with the criteria being identity > 75% and e-value < 1e-10. Matches that do not conform to chromosomal colinearity were removed manually.

A total of 2500 orthologs were common to all analyzed genomes and were used for constructing a genome-scale phylogenetic tree. For this, individual orthologous sequences were aligned by using MAFFT [24] and were then concatenated to obtain a “chromosomal” alignment; the phylogenetic tree was constructed with the program PHYML under GTR+gamma+I model [25].

Pseudogenes were identified by using the Psi-Fi perl script [26]. Briefly, genes that contain frameshifts, nonsense mutations, truncations or insertion/deletions (indels) that altered >20% of the amino acid sequence in comparison with the reference sequence were treated as pseudogenes. The exact site in which the pseudogenization event occurs was identified by using GeneWise software [27], which helps to align the pseudogene with its functional ortholog from the reference genome. Genes present in one *Shigella* strain but absent from the other *Shigella* strain were considered as a deletion event of the latter strain. If one gene is present in all the four *E. coli* strains but absent from the entire five *Shigella* strains, this gene is considered to be commonly decayed in all *Shigella* strains.

### Operon definition

The organization of operons in *E. coli* K12 MG1655 was taken from the database MicrobesOnline Operon Predictions [28] and RegulonDB [29]. Additionally, adjacent genes that have been proven to be involved in the same biological pathway (e.g., *thrL*, *thrA*, *thrB* and *thrC*) were considered to be of the same operon. A home-made Perl script was written for calculating the number of pseudogenes in their located operon.

### Functional assignment of *Shigella* decayed genes

COG (Cluster of Orthologous Groups of proteins) information was retrieved from NCBI COG database [30]. GenProtEC information was retrieved from *E. coli* genome and proteome databases [31]. We computed the number of genes for each function category and then used Chi-square test to detect which functional category differed significantly between decayed genes and functional genes.

### Ka/Ks analysis

The synonymous (Ks) and non-synonymous (Ka) substitution rates of the *Shigella* strains in comparison with the reference genome of *E. coli* K12 MG1655 were calculated by using codeml program in PAML package [32]. Genes were divided into three groups according the operon information. We did not compare Ka/Ks between groups directly in order to minimize the bias brought by the inherent differences between genes themselves; instead we compared ΔKa/Ks. CFT073 was used as the control, because this strain stays outside of all *Shigella* strains phylogenetically. For each orthologous gene, ΔKa/Ks  =  Ka/Ks*_Shigella_* – Ka/Ks_CFT073,_ in which Ka/Ks*_Shigella_* is Ka/Ks between a certain *Shigella* strain and K12, and Ka/Ks_CFT073_ is between *E. coli* CFT073 and K12.

### Expression potential of pseudogenes

The microarray-based transcription information of *S. flexneri* was retrieved from NCBI GEO database (eight sample records: GSM314300 – GSM314307). The "codon adaptation index" (CAI) values for *E. coli* K12 genes was calculated with codonW program (http://codonw.sourceforge.net/).

## Supporting Information

Table S1 Detailed list of decayed genes in the five Shigella strains.
(XLS)

Table S2 Detailed list of (A) genes commonly decayed in the five *Shigella* strains, and (B) genes decayed in at least one *Shigella* strain, and (C) genes in all decayed operon in *Shigella*.
(XLS)

Table S3 Recombination of pseudogenes in *Shigella*.
(XLS)

Table S4 Pseudogenes shared by SDY and O157.
(XLS)

Text S1 Comparison of recombination rate between *E. coli* and *Shigella*.
(DOC)

## References

[1] Kuo CH, Ochman H (2010). The extinction dynamics of bacterial pseudogenes.. PLoS Genet.

[2] Ochman H (2005). Genomes on the shrink.. Proc Natl Acad Sci U S A.

[3] Nilsson AI, Koskiniemi S, Eriksson S, Kugelberg E, Hinton JC (2005). Bacterial genome size reduction by experimental evolution.. Proc Natl Acad Sci U S A.

[4] Cole ST, Eiglmeier K, Parkhill J, James KD, Thomson NR (2001). Massive gene decay in the leprosy bacillus.. Nature.

[5] Fraser CM, Gocayne JD, White O, Adams MD, Clayton RA (1995). The minimal gene complement of Mycoplasma genitalium.. Science.

[6] Yang L, Jelsbak L, Marvig RL, Damkiaer S, Workman CT (2011). Evolutionary dynamics of bacteria in a human host environment.. Proc Natl Acad Sci U S A.

[7] Holt KE, Parkhill J, Mazzoni CJ, Roumagnac P, Weill FX (2008). High-throughput sequencing provides insights into genome variation and evolution in Salmonella Typhi.. Nat Genet.

[8] Scully LR, Bidochka MJ (2006). The host acts as a genetic bottleneck during serial infections: an insect-fungal model system.. Curr Genet.

[9] Maurelli AT, Fernandez RE, Bloch CA, Rode CK, Fasano A (1998). "Black holes" and bacterial pathogenicity: a large genomic deletion that enhances the virulence of Shigella spp. and enteroinvasive Escherichia coli.. Proc Natl Acad Sci U S A.

[10] Hale TL (1991). Genetic basis of virulence in Shigella species.. Microbiol Rev.

[11] Peng J, Yang J, Jin Q (2009). The molecular evolutionary history of Shigella spp. and enteroinvasive Escherichia coli.. Infect Genet Evol.

[12] Yang J, Nie H, Chen L, Zhang X, Yang F (2007). Revisiting the molecular evolutionary history of Shigella spp.. J Mol Evol.

[13] Peng J, Zhang X, Yang J, Wang J, Yang E (2006). The use of comparative genomic hybridization to characterize genome dynamics and diversity among the serotypes of Shigella.. BMC Genomics.

[14] Wei J, Goldberg MB, Burland V, Venkatesan MM, Deng W (2003). Complete genome sequence and comparative genomics of Shigella flexneri serotype 2a strain 2457T.. Infect Immun.

[15] Nie H, Yang F, Zhang X, Yang J, Chen L (2006). Complete genome sequence of Shigella flexneri 5b and comparison with Shigella flexneri 2a.. BMC Genomics.

[16] Yang F, Yang J, Zhang X, Chen L, Jiang Y (2005). Genome dynamics and diversity of Shigella species, the etiologic agents of bacillary dysentery.. Nucleic Acids Res.

[17] Jin Q, Yuan Z, Xu J, Wang Y, Shen Y (2002). Genome sequence of Shigella flexneri 2a: insights into pathogenicity through comparison with genomes of Escherichia coli K12 and O157.. Nucleic Acids Res.

[18] Hershberg R, Tang H, Petrov DA (2007). Reduced selection leads to accelerated gene loss in Shigella.. Genome Biol.

[19] Riley M, Abe T, Arnaud MB, Berlyn MK, Blattner FR (2006). Escherichia coli K-12: a cooperatively developed annotation snapshot--2005.. Nucleic Acids Res.

[20] Touchon M, Hoede C, Tenaillon O, Barbe V, Baeriswyl S (2009). Organised genome dynamics in the Escherichia coli species results in highly diverse adaptive paths.. PLoS Genet.

[21] Didelot X, Achtman M, Parkhill J, Thomson NR, Falush D (2007). A bimodal pattern of relatedness between the Salmonella Paratyphi A and Typhi genomes: convergence or divergence by homologous recombination?. Genome Res.

[22] Sharp PM, Li WH (1987). The codon Adaptation Index--a measure of directional synonymous codon usage bias, and its potential applications.. Nucleic Acids Res.

[23] Jansen R, Bussemaker HJ, Gerstein M (2003). Revisiting the codon adaptation index from a whole-genome perspective: analyzing the relationship between gene expression and codon occurrence in yeast using a variety of models.. Nucleic Acids Res.

[24] Katoh K, Toh H (2008). Recent developments in the MAFFT multiple sequence alignment program.. Brief Bioinform.

[25] Guindon S, Gascuel O (2003). A simple, fast, and accurate algorithm to estimate large phylogenies by maximum likelihood.. Syst Biol.

[26] Lerat E, Ochman H (2004). Psi-Phi: exploring the outer limits of bacterial pseudogenes.. Genome Res.

[27] Birney E, Clamp M, Durbin R (2004). GeneWise and Genomewise.. Genome Res.

[28] Price MN, Huang KH, Alm EJ, Arkin AP (2005). A novel method for accurate operon predictions in all sequenced prokaryotes.. Nucleic Acids Res.

[29] Gama-Castro S, Jimenez-Jacinto V, Peralta-Gil M, Santos-Zavaleta A, Penaloza-Spinola MI (2008). RegulonDB (version 6.0): gene regulation model of Escherichia coli K-12 beyond transcription, active (experimental) annotated promoters and Textpresso navigation.. Nucleic Acids Res.

[30] Tatusov RL, Fedorova ND, Jackson JD, Jacobs AR, Kiryutin B (2003). The COG database: an updated version includes eukaryotes.. BMC Bioinformatics.

[31] Serres MH, Goswami S, Riley M (2004). GenProtEC: an updated and improved analysis of functions of Escherichia coli K-12 proteins.. Nucleic Acids Res.

[32] Yang Z (2007). PAML 4: phylogenetic analysis by maximum likelihood.. Mol Biol Evol.

