# Supplementary material for: Gene Decay in Shigella as an Incipient Stage of Host-Adaptation
Source: PLoS One. 2011 Nov 16;6(11):e27754. doi: 10.1371/journal.pone.0027754 (PMC3218036; doi:10.1371/journal.pone.0027754)
Supplement: Text S1 — Comparison of recombination rate between E. coli and Shigella. (DOC) [file pone.0027754.s005.doc]

# Supporting Text:

# Recombination rate for *Shigella* and *E. coli*

**Materials and methods**

Whole genome alignments of the 9 *E. coli/Shigella* strains were performed using MAUVE program [1]. The coordinates of all backbone segments on each genome were extracted from the MAUVE output file “backbone” and aligned with MAFFT [2]. Individual alignments were concatenated, and the length of the total alignment was 3,035,646 bp.

We split the alignment into two groups: one includes the four *E. coli* strains, and the other includes the five *Shigella* strains. The program SITES was used for calculating mutation and recombination rate [3], with window size 3 kbp and step size 0.5 kbp.

**Results**

The average recombination rate (per bp) is 0.056 and 0.013 for *E. coli* and *Shigella*, respectively. The average ratio of recombination rate to mutation rate (c/u) is 2.718 and 0.921 for *E. coli* and *Shigella*, respectively.

Figure 1 in this document illustrates the comparison of recombination rate between *E. coli* and *Shigella*.

Paired T test shows that both recombination rate and c/u in *E. coli* are significantly larger than that in *Shigella* (p value < 0.001).


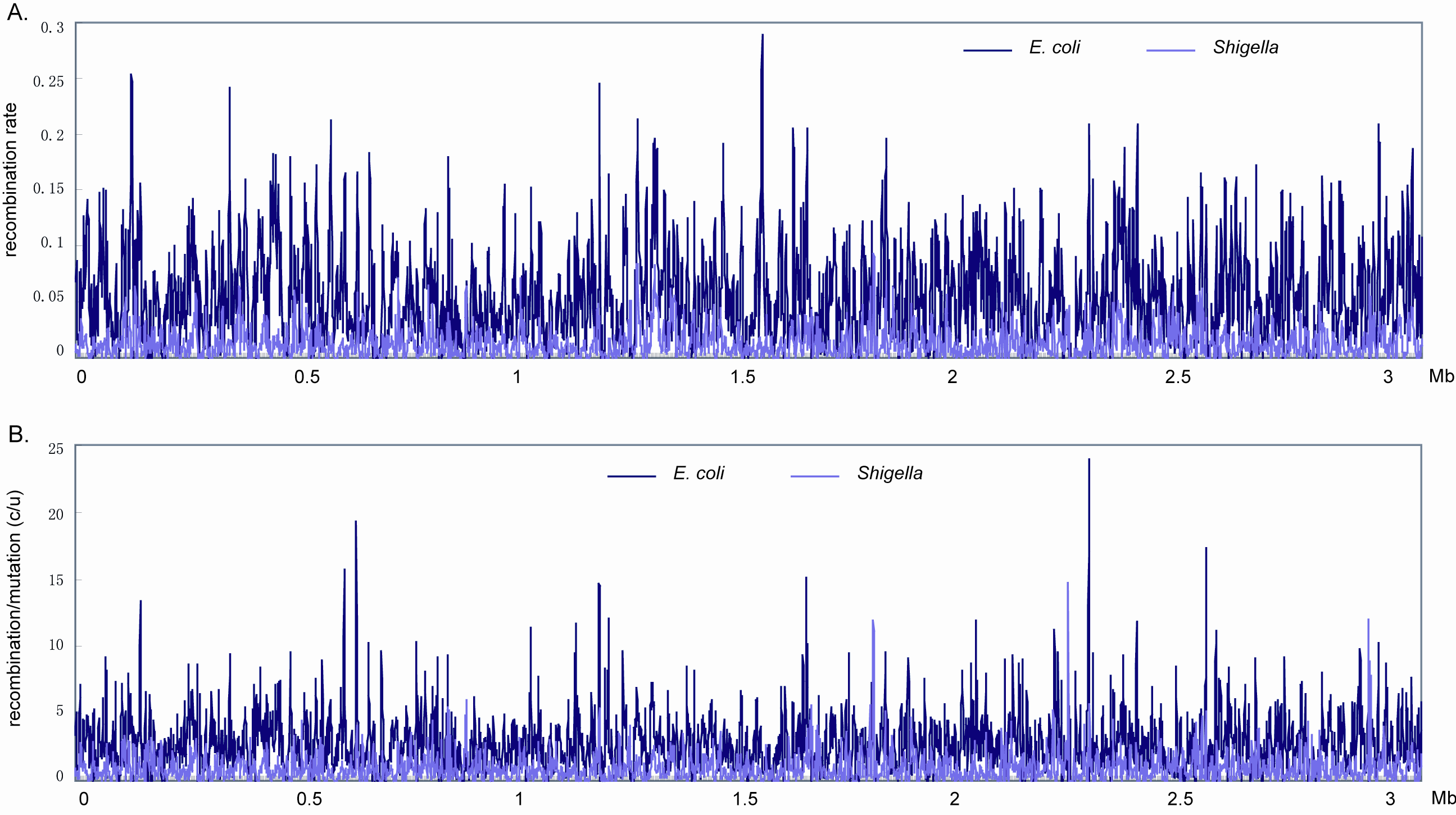


**Fig. 1. Comparison of recombination rate between *E. coli* and *Shigella*.** (A) Recombination rate (per bp). (B) Ratio of recombination rate to mutation rate.

**References**

1. Aaron C.E. Darling, Bob Mau, Frederick R. Blatter, and Nicole T. Perna: **Mauve: multiple alignment of conserved genomic sequence with rearrangements.** *Genome Research* 2004, 14(7):1394-1403.

2. Katoh K, Kuma K, Toh H, Miyata T: **MAFFT version 5: improvement in accuracy of multiple sequence alignment**. *Nucleic Acids Res* 2005, 33: 511–518.

3. Hey J, Wakeley J: **A coalescent estimator of the population recombination rate**. *Genetics* 1997, 145:833-46.
